# Association between Maternal Periodontitis and Development of Systematic Diseases in Offspring

**DOI:** 10.3390/ijms23052473

**Published:** 2022-02-24

**Authors:** Anna Starzyńska, Piotr Wychowański, Maciej Nowak, Bartosz Kamil Sobocki, Barbara Alicja Jereczek-Fossa, Monika Słupecka-Ziemilska

**Affiliations:** 1 Department of Oral Surgery, Medical University of Gdańsk, 7 Dębinki Street, 80-211 Gdańsk, Poland; b.sobocki@gumed.edu.pl; 2 Department of Oral Surgery, Medical University of Warsaw, 6 Binieckiego Street, 02-097 Warsaw, Poland; piotrwychowanski@wychowanski.pl; 3 Specialized Private Implantology Clinic Wychowanski Stomatologia, 9/33 Rakowiecka Street, 02-517 Warsaw, Poland; 4 Department of Periodontology and Oral Diseases, Medical University of Warsaw, 6 Binieckiego Street, 02-097 Warsaw, Poland; mattianow@gmail.com; 5 Department of Oncology and Hemato-Oncology, University of Milan, 7 Festa del Perdono Street, 20-112 Milan, Italy; barbara.jereczek@ieo.it; 6 Division of Radiotherapy, IEO European Institute of Oncology, IRCCS, 435 Ripamonti Street, 20-141 Milan, Italy; 7 Department of Human Epigenetics, Mossakowski Medical Research Institute, Polish Academy of Sciences, 5 Pawińskiego Street, 02-106 Warsaw, Poland; mslupecka@imdik.pan.pl

**Keywords:** maternal periodontitis, periodontitis, gingivitis, periodontal disease, periimplantitis, pregnancy complication, epigenetic programming, offspring, systemic diseases

## Abstract

Periodontal disease (PD) is one of the most common oral conditions affecting both youths and adults. There are some research works suggesting a high incidence of PD in pregnant women. As an inflammatory disease of bacterial origin, PD may result in the activation of the pathways affecting the course and the pregnancy outcome. The authors, based on the literature review, try to answer the PICO question: Does maternal periodontitis (exposure) influence the incidence of complications rates in pregnancy and the development of systemic diseases in childhood and adult offspring (outcome) in the humans of any race (population) compared to the offspring of mothers with healthy periodontium (comparison)? The authors try to describe the molecular pathways and mechanisms of these interdependencies. There is some evidence that maternal periodontitis may affect the pregnancy course and outcome, resulting in preeclampsia, preterm delivery, vulvovaginitis and low birth weight. It can be suggested that maternal periodontitis may affect offspring epigenome and result in some health consequences in their adult life.

## 1. Epidemiology of Periodontal Disease (PD)

The findings show that oral conditions remain a substantial population health challenge affecting 3.9 billion people at the beginning of the second decade of 21st century. The global burden of oral conditions has been shifting from severe tooth loss in recent decades toward severe periodontitis and untreated caries. Severe periodontitis and untreated caries in deciduous teeth were the 6th and 10th most prevalent oral conditions, affecting, respectively, 11% and 9% of the global population [1].

Dental caries and periodontitis are the most common oral diseases, and major causes of tooth loss. Recently, we have observed a decrease in the incidence of caries in the global population, but not in the incidence of severe periodontitis. In 2010, severe periodontitis affected 743 million people aged 15–99 worldwide, while the age-standardized incidence of severe periodontitis did not change between 1990 and 2010, being 701 cases per 100,000 person years in 2010 [2]. Periodontal diseases are very common and are supposed to affect up to 90% of the world’s population in the course of their individual lives [3].

The total cost of performance loss from severe periodontitis is estimated at $54 billion annually, while the overall economic impact of periodontal disease is an important component, direct and indirect, of oral disease costs of $442 billion in 2010 [4].

Periodontitis is known as a complex infectious disease, and may begin in childhood or adolescence, but most of all, clinically manifests itself in early adulthood, and less often, in later years. It is characterized by primary reversible and than permanent histopathological disorders. Clinically periodontitis appears primary as the inflammation process of soft tissue (gingivitis), followed by hard tissue alterations like alveolar bone loss and dental cementum pathologies. If not treated properly, this condition leads to increased tooth mobility due to the loss of tooth attachment apparatus, as a result of the destruction of periodontal ligaments (PDL), and ultimately tooth loss. All the pathology is accompanied with the local tissue inflammation process [5,6].

In light of contemporary literature, periodontopathies are among the most common inflammatory conditions in humans, similar to diabetes and obesity as social diseases. Periodontal diseases, due to their prevalence, can also be considered social diseases. The epidemiological studies carried out in Poland, commissioned by the Ministry of Health, showed that among children and adolescents aged 12 years, healthy periodontium was found only in 47.3% of the respondents; in the group of 18-year-old adolescents, healthy periodontitis was found in 37.3% of patients, and advanced disease periodontitis was present in 0.5% of adolescents. On the other hand, in the group of adult Poles aged 35–44, only 1% of patients have healthy periodontitis, and more than 16% of people are diagnosed with advanced periodontitis [7].

### Periodontal Disease (PD) Is a Serious Problem in the Pregnant Women Population

Pregnancy may be associated with an increased risk of periodontal disease. The prevalence of periodontitis was found to be very high in some groups of pregnant woman, and may even score as high as 66.7% in some ethnic groups, even in highly developed societies [8,9].

The numerous studies reported that knowledge about periodontal disease and its potential effects on pregnancy is strongly limited in the population of pregnant women. The study of Asa’ad et al. showed that, in Saudi Arabia, only 12% of respondents knew about the connection [10]. Factors such as age, education, or even marital status may influence this knowledge [11]. In the French population, only 10.5–18% of women consulted, during pregnancy, oral health topics with medical professionals. There was a positive correlation between dental consultations before pregnancy and the lack of dental examination during that period [12,13].

Although some women have knowledge about the relation of PD to adverse pregnancy outcomes, only 47% of them received a diagnosis of oral health or appropriate treatment [12]. All of these data suggest that there is a need for the education of women in the pregnancy period about PD and its consequences, due to general poor knowledge [14,15].

According to the Center for Disease Control and Prevention in the U.S., the incidence of PD is 47.2% in the population of adults aged 30 and older. The PD is more common in older, poor, male, less than high school educated, and currently smoking patients (man vs. woman: 56.4% vs. 38.4%) population [16]. Taking this into consideration, there is still a serious danger that many pregnant women are at risk of PD consequences. Moreover, González-Jaranay proved that all parameters which describe PD, such as plaque index, probing death or gingival index, increase during the gestation period [17].

Other pregnancy-related states such as diabetes mellitus may also enhance that risk, interacting with PD. Several studies showed that periodontal inflammation, destruction, and their impact on systemic diseases are stimulated by the presence of diabetes, and that diabetes may increase the severity of PD consequences [18,19,20,21,22]. For this reason, the prevention of consequences by the implementation of dental routine control during pregnancy should be considered, in addition to measuring the glycemia level.

The numerous studies indicated some adverse pregnancy outcomes related to PD, such as preeclampsia, premature rupture of the membrane (with associated preterm delivery and vulvovaginitis), and low birth weight (fetal growth restriction) [23,24,25,26]. There are also some less evidenced and indicated, but not currently confirmed states, like intra-amniotic inflammation caused by PD [23].

## 2. The Aim of the Review

The authors following the PICO (PICO is derived from the acronym for: Patient/Population, Intervention, Comparision, Outcome) guidelines want to answer, in this narrative review, the clinical question raised from the presented background data [27]. Does maternal periodontitis (intervention/exposure) influence the incidence of complications rates in pregnancy and the development of systemic diseases in childhood and adult offspring (outcome) in humans of any race (population), compared to the offspring of mothers with healthy periodontium (comparison)?

## 3. The Clinical Picture and Etiology of Periodontitis

The vast majority of periodontal diseases are classified as bacterial inflammations, initially affecting the gingival tissue, and then, as the disease progresses: periodontium, alveolar bone, and tooth root cementum. The initial symptoms are typical of gingivitis, and include the appearance of gingival pockets, bleeding gums, redness, swelling, fluffiness, and unpleasant smell from the mouth. Other symptoms that may appear are typical for periodontitis, i.e., the presence of periodontal pockets, destruction of the connective tissue attachment, periodontium, alveolar bone, the lengthening of clinical crowns of teeth, tooth movement, their loosening, and, finally, loss [3,28].

The main cause of periodontal disease is dental biofilm, dominated by G (-) bacteria, and *Porphyromonas gingivalis*, *Treponema denticola*, *Agregatibacter actinomycetemcomitans* are of particular importance in periodontitis [4,29]. Periodontitis is a chronic or acute condition of the tissues surrounding the tooth of complex etiology, which is the cause of the local and general increase in inflammatory mediators and immune complexes, such as C-reactive protein (CRP), fibrinogen, nitric oxide (NO), interleukin 1 (IL-1), interleukin 6 (IL-6), and tumor necrosis factor (TNF-α) [30].

Periodontitis is not a natural consequence of untreated gingivitis, but it develops as a result of the pathogen–host immune system imbalance, as a result of the weakening of the immune system of patients due to, e.g., old age, genetic factor, smoking, diabetes, stress, osteoporosis, improper eating habits, AIDS, and other comorbidities [29,31,32].

Gingivitis is a non-specific reaction to an increase in the number of bacteria (both Gram-positive and Gram-negative) in/or under the gingival groove. In contrast, periodontitis is associated with the multiplication of a certain species of gram-negative bacteria in the gingival furrows. The result is the formation of a periodontal pocket, which favors the even greater accumulation of bacteria, and changes the composition of the microbiota. Supragingival plaque (biofilm) in people with healthy gums consists of several–a dozen (1–20) layers of Gram-positive cocci (*Streptococcus mutans*, *S. mitis*, *S. sanguis*, *S. oralis*, *Rothia dentocariosa*) and Gram-positive cells bacilli (*Actinomyces viscosus*, *A. israelii*, *A. gerencseriae*, *Corynebacterium* spp.), and a few Gram-negative cocci (*Veillonella parvula*, *Neisseria* spp.). Biofilms occurring in people with periodontitis are most often subgingival, characterized by a complex multi-layered structure. The composition of the bacterial population in the active, aggressive phase of the disease differs from that in the period of remission. The dominance of the species *Tannerella forsythia*, *Treponema denticola*, *Porphyromonas gingivalis*, *Prevotella intermedia* and *Campylobacter rectus* is associated with a depth of periodontal pockets greater than 4 mm, and bleeding when examined with a periodontal probe. Apart from the mentioned species, the presence of other gram-negative anaerobes such as *Aggregatibacter actinomycetemcomitans*, *Fusobacterium nucleatum*, and *Eikenella corrodens* can be found [33,34,35,36].

Research was conducted into medical databases: PubMed and Scopus, for studies on periopathogens occurring in various populations of people, with severe periodontal disease, mild periodontal disease, and healthy people. The condition of the periodontium was assessed on the basis of a clinical examination (PD–pocket depth, BoP–bleeding on probing, PI–plaque index, CAL–clinical attachment loss) for clinical and radiological examination. The identification of bacterial genetic material was performed by PCR tests [31,37].

In a 2018 study of patients with periodontal disease in the Congo, the presence of *P. gingivalis*, *T. forsythia*, *T. denticola* was found to be strong, and *P. intermedia* accounted for 75% of the red complex. However, the presence of *A. actinomycetemcomitans* was not detected. *P. intermedia*, belonging to the orange complex, has been frequently detected. A correlation between the periodontal disease symptoms and the prevelence *of P. gingivalis*, *T. denticola*, *T. denticola*, *T. forsythia* in subgingival plaque was very potent [38].

Another large study conducted in 2012–2016 in Saudi Arabia of 2435 school-age 12–16-year-olds showed active periodontal disease in 209 of these subjects, and in 69 subjects with completely healthy periodontium as a control group. Overall, 57.4% of the respondents had at least one type of bacteria, at least two types in 19.1%, and only 9.0% had at least three types of bacteria, and 1.8% had at least four types of bacteria. A statistically significant correlation was found between the young age of the respondents and the presence of *A actinomycetemcomitans* [39].

## 4. Periodontal Disease in Pregnancy

The whole-body inflammatory pre-activation associated with untreated periodontitis is a serious risk factor for numerous systemic diseases, such as: atherosclerosis, acute coronary events, strokes, diabetes, obesity, Parkinson’s disease, asthma, and erectile dysfunction, but also low birth weight and premature births [7,32,40,41].

Bacteremia is defined as the transient or continuous presence of viable bacteria in the bloodstream. In periodontitis, the pathogenic subgingival microflora is in close contact with the damaged internal epithelium of the periodontal pockets, which allows pathogenic bacteria to enter the bloodstream. Chronic low-level bacteremia is believed to be the direct mechanism explaining the association between an undesirable course of pregnancy and periodontitis [42].

The mechanism of biological activity of perio-pathogens during pregnancy is directly related to the microbes of the cavity and their products (endo and exotoxins), which get into the circulatory system and reach the fetoplacental unit. The indirect mechanism relates to the influence of inflammatory mediators produced locally in periodontal tissues, which also reach the fetal–placental unit and the liver through the bloodstream, and increase systemic inflammation through the reaction of the acute phase protein, C-reactive [43].

In 2012, the strongest evidence was judged to be of a direct mechanism for the hematological spread of microorganisms from the oral cavity and their products, which then induce an inflammatory and immune response in the feto-placental unit. In addition, it has been proposed that the presence and level of microorganisms and their products in the amniotic fluid, placental umbilical cord blood, neonatal respiratory aspirates, placenta, fetal membranes, or fetal tissues, along with the levels of antibodies to oral microbes, are the best materials for assessing exposure to oral bacterial infections in the oral cavity fetal–placental unit. Of all the bacteria species, *Fusobacterium nucleatum*, *Campylobacter rectus*, *Porphyromonas gingivalis* and *Bergeyella* sp. turned out to be the most strongly associated with abnormalities in pregnancy. On the other hand, levels of inflammatory biomarkers such as IL-1, IL-6, prostaglandin-E2, TNF-α, CRP, 8-isoprostane, soluble intercellular adhesion molecule-1, matrix metalloproteinases, fibronectin, and α-fetoprotein) in maternal serum, umbilical cord blood, and amniotic fluid were considered the best parameters for a potential assessment of the exposure to inflammation of the fetoplacental unit [44].

The first evidence of a direct relationship between the negative influence of the oral microbiome on the uterine microbiome was described in 2006. *Bergeyella* was established as the only infectious agent in the amniotic fluid of a woman with an infection leading to premature birth (at 24 weeks of pregnancy). Further analysis showed an identical bacterial strain in the mother’s subgingival plaque, while the bacterial flora of her vagina was not detected [45]. Four studies were included, demonstrating the positive effect of periodontal treatment on inflammatory parameters at gingival crevicular fluid levels, and some from blood serum, but not at the level of the umbilical cord. Therefore, to elucidate the mechanisms linking periodontitis and spontaneous preterm birth, further periodontal intervention studies, including the quantitative and qualitative analyses of proinflammatory cytokines and chemokines in samples, harvested from gingival crevicular fluid, maternal blood, and cervicovaginal fluid/amniotic fluid, are required [46,47]. Since *Bergeyella* is a common species of bacteria in the oral cavity, and is absent in the vaginal flora, it should be considered a strong marker of oro-uterine infectious transmission.

*Fusobacterium nucleatum* is another species of oral bacteria that is often associated with an undesirable course of pregnancy. It is also absent in the physiological flora of the vagina. The incidence of its appearance is inversely proportional to the gestational age at delivery [48,49,50]. *Fusobacterium nucleatum* has been identified in various areas of the placenta and fetal tissues, such as amniotic fluid, amniotic membranes, umbilical cord blood, and neonatal gastric aspirates, when adverse events are identified during pregnancy. It was the only infectious agent, or was present in mixed infections [44,46,47].

Physiological changes involving inflammatory pathways occur in the reproductive organs before delivery, even in the case of full-term deliveries without clinical signs of infection. Hormonal changes at the end of pregnancy induce the release of pro-inflammatory cytokines into the maternal serum, which in turn initiates the production of prostaglandins in the myometrium, which can lead to the contraction of the uterus. Such pro-inflammatory cytokines include IL-6, interleukin-8 (IL-8), and Interleukin-1β (IL-1β) [51,52,53,54].

Pro-inflammatory mediators secreted from inflammatory cells in the periodontium to the blood vessels are delivered systemically to other tissues and organs. Thus, it has been hypothesized that the physiological cascades that initiate labor can also be activated indirectly by inflammatory mediators produced and disseminated from the periodontium [55].

Studies were carried out on the effect of periodontal treatment during pregnancy on the level of pro-inflammatory cytokines contained in the gingival fluid in the peripheral blood plasma, in relation to the course of pregnancy. The concentrations of IL-1β, IL-6, IL-8, interleukin-10 (IL-10), interleukin-12p70 (IL-12), interleukin-17A, chemokine-2 ligand/monocyte chemoattractant-protein-1 (CCL2/MCP1) and TNF- α were analyzed. It was shown that active periodontal treatment significantly reduced the levels of IL-1, IL-10, IL-12, and IL-6 in the gingival fluid at 28 weeks of gestation, compared to the control group. However, no significant difference in the course of pregnancy was observed between the treated women and the control women. In addition, levels of gingival fluid, CCL2/MCP1, IL-8, and TNF-α were significantly increased. Following periodontal intervention, significant reductions in periodontal clinical variables and gingival fluid levels of TNF-α and IL-6 were observed, but no significant changes were observed in any of the serum biomarkers [56,57].

## 5. Epigenetic Regulations in the Pathophysiology of Periodontal Disease

Periodontitis is a complex, multifactorial disease. Multiple conditions such as environmental factors (microbial biofilm), lifestyle (diet, smoking, stress), and general health (diabetes mellitus) may modify host immune response, and thus contribute to the development of periodontitis. Nowadays, the increasing number of evidence suggests that the route through which internal and external environments may regulate biologically relevant processes involved in the etiology of complex diseases are epigenetically affected. Epigenetic is used to describe processes that lead to heritable changes to the activity of genes without changes in the DNA sequence [58]. Changes to the epigenome appear at different organizational units, including DNA methylation, histone modification, and non-coding RNA, although non-coding RNAs are not included in epigenetic mechanisms by some specialists, since they interfere on the posttranscriptional level. While the contribution of environmental exposures to the pathobiology of periodontitis is widely recognized [59], an explanation of how these translate into epigenetic effects in the context of periodontitis is still being investigated [60].

### 5.1. DNA Methylation

DNA methylation is the most extensively studied epigenetic mechanism. During DNA methylation, the covalent transfer of a methyl group occurs in the 5′ position of cytosine residues. Methylated cytosines are found primarily at the cytosine-guanine dinucleotides (CpG), but are also observed at non-CpG sites (CpA, CpT, and CpC). In general, methylation causes chromatin condensation and the disruption of interactions between DNA and transcription factors, which are associated with transcriptional repression. Cytosine residues are methylated by DNA methyltransferases (DNMTs), and can undergo passive or active demethylation. Passive DNA demethylation occurs in the course of replication due to the “dilution” of methylation marks. Active demethylation requires ten–eleven translocation (TETs) enzymes, and occurs through the excision of 5-formylcytosine (5fC) or 5-carboxylcytosine (5caC), by the thymine DNA glycosylase (TDG) and base-excision repair (BER) pathway.

In patients suffering from periodontal disease, the alterations in the methylation of the gene promoter were observed for several genes coding peptides associated with inflammatory tissue responses (cytokines, chemokines), and for receptors, signaling the molecules and transcription factors involved in periodontitis. The abovementioned changes were observed in the gingival tissue, as well as in the peripheral blood and buccal mucosa from patients with periodontitis [61].

In some studies, reported changes in DNA methylation in periodontitis were supported by studies showing their relationship with gene expression. Negative correlations between the levels of DNA methylation at gene promoter regions and gene expression have been found for *TNF* [62], *PTGS2* [63], *TLR2* [64], and *IFNG* [65] in gingival biopsies, and for IL8 in oral epithelial cells [66]. In blood samples, the promoter methylation of *TNF* [67], but not IL6 [68], inversely correlates with serum protein levels.

Recent studies have found differences in the expression of DNMTs, both in periodontal cells and different primary cell types, in response to specific factors involved in periodontitis pathogenesis [61]. Moreover, in the study where response to inflammatory stimulation was investigated in the gingival fibroblasts, the downregulation of TET1 was observed [69].

In another study, TET2 silencing resulted in reduced cytokine expression in dental pulp cells after LPS stimulation, associated with decreased *MYD88* gene promoter hydroxymethylation levels, and diminished NF-κB signaling [70].

These studies suggest that TET enzymes regulate immune response, and the mechanism of their action can be independent of DNA demethylation.

It should also be mentioned that environmental risk factors for periodontitis such as smoking, obesity, and diabetes (including gestational diabetes mellitus) significantly influence DNA methylation, as well as the activation of pro-inflammatory pathways.

### 5.2. Histone Modifications

Histone modifications (HM) play a significant role in cell biology, modulating the regulation of gene expression and many further processes [71]. Being such an important regulator, HM are present in many diseases or conditions like cancer, obesity, and diabetes, as well as PD and pregnancy outcomes [72,73,74,75]. Although a direct association between histone modifications and the PD or adverse pregnancy outcomes was reported, looking for a potential link may be challenging, due to the lack of analysis of both conditions in one model available in the literature.

It was reported that dysbiosis that drives PD may lead to epigenetic modifications like the acetylation of histones. It was also proven that the in vitro exposure of oral epithelial cells to LPS might result in histone modifications, the activation of coactivators (e.g., p300/CBP), and an NF-κB increase. Moreover, Toll-like receptors 1,2, and 4 activated by pathogens also induce histone acetylation [76]. Knowing that PD can enhance systemic immune response and may change the cytokine profile in blood [77], HM seems to be one of the factors inducing this process. The data from cancer studies revealed that HM are used by cancer to normalize the impaired immunosurveillance and trigger an antitumor immune response, which probably support the relevant role of HM in the modulation of immunological processes [78], also in the presumable connection between PD and pregnancy adverse outcomes (e.g., low birth weight, preterm birth) [77]. Further and detailed studies investigating this mechanism should be conducted. The study of Francis et al. identified the histone lysine methyltransferase SETD1 as the key agent in the opening of the chromatin on inflammatory gene promoters through histone H3K4-mediated trimethylation [79]. On the other hand, the study of Cantley et al. showed that the inhibition of both Class I and Class II histone deacetylases had no impact on inflammation in mice [80].

Concerning pregnancy adverse outcomes and epigenetics, studies mainly describe the role of DNA methylation and miRNAs in the development of fetal alcohol spectrum disorders. Due to worse stability and the poor characterization of HM in this disease having not been well researched [81]. Interesting results were provided by studies describing drug interaction with protein acetylation. For example, using trichostatin ex vivo preserved, by possible epigenetic mechanism, progesterone receptor inactivation, and reduced contractility by increasing heat shock protein 20 acetylation, which promoted actin depolymerization and relaxation [82].

Although some associations may be observed, the mechanism of connection between PD and pregnancy adverse outcomes is still undiscovered. The role of epigenetics should be thoroughly evaluated in a common model combining PD and pregnancy conditions.

### 5.3. Non-Coding RNAs

Non-coding RNAs (ncRNAs) are RNA sequences making up to 98% of the human transcriptome. NcRNAs do not code proteins, but they are found to participate in multiple biological processes, and regulate physiological and developmental processes and disease. The epigenetic noncoding RNAs (ncRNAs) can be divided, considering their roles as the housekeeping ncRNAs (ubiquitously expressed in cells, primarily regulate generic cellular functions) and regulatory ncRNAs (regulators of gene expression at epigenetic, transcriptional, and post-transcriptional levels). Regulatory ncRNAs could be further classified as small non-coding RNAs (sncRNAs) with comprising transcripts fewer than 200 nt, and lncRNAs with lengths greater than 200 nt. The main classes of small ncRNAs are microRNA (miRNA), small interfering RNAs (siRNAs), and piwi-interacting RNAs (piRNAs). However, some ncRNAs, such as circular RNAs, due to their variable length, might belong to both classifications at the same time [83].

There are several studies investigating ncRNAs in periodontal disease, with the focus on their use as new diagnostic tools. However, it is worth paying attention to their role in the pathogenesis and regeneration of the disease itself.

### 5.4. MicroRNAs

MicroRNAs (miRNAs) are a group of small noncoding RNAs of about 22 bp in length that regulate gene expression through post-transcriptional modifications, by binding to the 3′-untranslated region of a target messenger RNA (mRNA), which leads to the suppression of gene expression, either by the degradation of a target mRNA, or by prevention of its translation. Interestingly, one miRNA can control the expression of several genes, whereas the expression of a certain gene can be controlled by several miRNAs [84].

MicroRNAs are considered as epigenetic mechanisms that modulate cellular processes, such as cell growth, apoptosis, and differentiation, and play fundamental roles in inflammatory responses and the development of diseases, e.g., cancer and systemic rheumatic diseases [85,86]. Moreover, microRNAs have recently emerged as key regulators in bone hemostasis [87].

Recently, the expression of miRNAs in tissues affected by periodontitis and the interaction between miRNAs and *Porphyromonas gingivalis* have been extensively investigated. It was reported that miRNAs might mediate endotoxin tolerance through the modulation of the mitogen-activated protein kinase (MAPK) signaling pathway, increase the sensitivity of Toll-like receptors (TLRs) when exposed to bacterial lipopolysaccharide (LPS), or target the nuclear factor-kappa B (NF-κB) signaling pathway in response to bacterial stimuli [88].

Results from a recent meta-analysis of human studies (43 articles included, 563 miRNAs identified, 16miRNAs selected for meta-analysis) show an overall upregulation in the expression of the most evaluated miRNAs in periodontitis, in comparison to healthy periodontium [89]. On the basis of both the microarray and RT-PCR values, the role of miRNA-146 in the regulation of innate immune response was outlined. An overexpression of miRNA-146 in patients with rapidly progressive aggressive periodontitis was shown to be accompanied by a reduction in the levels of TNF-α, IL-1β, and IL-6, which suggests the existence of a negative feedback loop between mi-146 and pro-inflammatory cytokine synthesis [90]. It is worth mentioning that changes in the expression of miRNA-146 have previously been associated with several inflammatory disorders [90,91,92]. Other miRNAs significantly upregulated in periodontitis were miRNA-223, which is a key regulator of osteoclastogenesis and miRNA-30e, miRNA-130a, miRNA-142-3p, and miRNA-210. All four of them were found to be overexpressed in the presence of periodontitis and obesity [91].

Moreover, in the experimental models of periodontitis miRNA-155, expression was significantly upregulated. The mir-155 family is widely involved in immune adjustment [93] and a variety of cancers [94]. Studies by Mahesh et al. [95] using GO analysis for target genes showed genes cebpb and VCAM1 and the protein SMAD2, which might be the key factors in mir-155-regulated immune activities. SMAD2 plays a very important role in the regulatory network of mir155 target genes and immune-related proteins. As known, SMAD2 proteins are signal transducers and transcriptional modulators that mediate multiple signaling pathways and are responsible for the transmission of extracellular signals from ligands of the transforming growth factor beta (TGF-β) superfamily of growth factors into the cell nucleus [96,97].

Understanding the functional roles of miRNAs in the pathogenesis, progression, and regression of periodontitis has strong therapeutic potential. miRNA therapeutics hold great promise for the future of periodontal therapy, based on their ability to modulate the immune response to infection.

### 5.5. Long Noncoding RNAs

Most lncRNAs have been studied in relation to processes such as carcinogenesis, osteogenesis, diabetes, neurological diseases, and cardiovascular diseases [98], suggesting their important role as regulators in the interaction between pathogens and hosts. Emerging evidence has suggested that long non-coding RNAs (lncRNAs) are linked to alterations in immune-related genes and cell subpopulations, including regulating T cells through FOXP3 [99], controlling dendritic cell differentiation through STAT3 binding to lncDC [100], and activating the differentiation of CD4+ and CD8+ T cells [101]. Their involvement in immune response make them valuable diagnostic, prognostic, and therapeutic targets for several inflammatory diseases. The involvement of lncRNAs in the pathophysiology of periodontitis was described in detail by Xu et al. [102]. Moreover, a microarray-based analysis of lncRNA signature in periodontitis revealed a differential expression of hundreds of lncRNAs, between affected and adjacent normal tissues [103].

Long non-coding RNAs can be localized in the nucleus or cytoplasm. Depending on the subcellular localization of the lncRNAs, they directly regulate gene expression by binding to chromatin regulatory proteins, which influence chromatin modification. They can also regulate mRNA or interact with other RNAs and proteins on transcriptional and post-transcriptional levels.

Previous microarray studies [104] were confirmed by the high-throughput sequencing method, revealing an existence of the competing endogenous RNAs (ceRNA) interaction network of lncRNA-miRNA-mRNA, which may be involved in the regulation of the pathogenesis of periodontitis [105].

MicroRNA and lncRNA are detected in tissue samples, as well as in all body fluids, such as blood (plasma/serum), urine, cerebrospinal fluid, tears, and saliva. ncRNAs, especially microRNAs and lncRNAs, are very stable, even in RNAse-rich biofluids, thus making them potential new biomarkers [106]. Moreover, they are also identified in exosomes (the smallest subtype of extracellular vesicles), new mechanisms of cell-to-cell communication. Secreted exosomes circulate in different body fluids, and they can provide autocrine, paracrine, and endocrine signaling [107,108]. Special attention should be paid to the role of salivary exosomes in the mechanisms of linking periodontitis with systemic diseases.

## 6. The Maternal Periodontal Disease in Pregnancy and Consequences in Offspring

There is an increasing amount of evidence suggesting a systemic link between maternal periodontal disease in pregnancy and adverse health consequences in offspring. Among them, there are short-term and long-term effects. The short-term consequences of maternal periodontitis include smaller fetuses [109], fetal loss [110], preterm delivery [110,111,112,113,114,115], preeclampsia [116,117], and small for gestation aged infants [110]. However, this parameter could be the first sight of long term effects, as the low birth weight may be associated with an increased incidence of adult-onset cardiovascular and metabolic diseases, such as hypertension, coronary heart disease, obesity, and type 2 diabetes, as well as greater risk of reproductive and neurological disorders in later life [118,119].

According to animal research using experimentally induced maternal periodontitis, long-term effects of periodontal disease during pregnancy include insulin resistance [120], effects on the central nervous system development [121], and the increased risk of development of lung inflammatory allergic response in the offspring [122]. These happen due to a phenomenon known as programming, which can translate changes in the intrauterine microenviroment into physiologic and metabolic alterations in adulthood. The mechanisms by which diseased periodontal tissues in the mother may influence offspring health seems to be very complex processes. The following searching procedure was implemented in assessing the current state of knowledge regarding the association between maternal periodontitis and offspring health. We searched PubMed using “maternal periodontitis AND offspring” and “maternal periodontal disease AND offspring” as keywords. These searches resulted in 29 and 26 original articles, respectively. After the verification of both searches, we selected 14 original articles written in English, which were related to the topic and included in this paper.

So far, published results show that maternal periodontitis in pregnancy influences a developing fetus via different mechanisms and pathways.

### 6.1. Maternal Periodontitis as a Chronic Exposure to Inflammatory Mediators

One of them is the effect of chronic exposures on inflammatory mediators which may directly affect the development of the fetus. Significant increases in serum levels of TNF-α, and IL-6 were reported in maternal animals with ligature-induced periodontitis, as well as in offspring exposed to maternal periodontitis in the pregnancy and lactation period [122]. Increased levels of TNF-α and IL-6 were also reported in human mothers with periodontitis [123].

It was previously reported that IL-6, a proinflammatory cytokine, leads to the development of insulin resistance and T2DM via the involvement of various pathways: induces the expression of suppressor of cytokine signaling 3 (SOCS-3), which inhibits insulin signaling, and participates in the JAK/STAT transcription pathways and the STAT3 phosphorylation tyrosine phosphorylation of IRS-1 and -2. Moreover, some animal and in vitro studies revealed that chronic exposure to proinflammatory cytokines (IL-6, IL-1β, IFN-γ and TNF-α) potentiates the reduction of β-cell mass and disrupts the function of β-cells, leading to impaired glucose-stimulated insulin secretion [124,125]. These results correspond with animal study by Mattera et al. [126], showing that an increase in pro-inflammatory cytokines in the course of periodontitis results in insulinemia, insulin resistance, reduced GLUT4 content in the plasma membrane, translocation index after insulin stimulation, and decreased Akt serine phosphorylation status in offspring. Moreover, an increased level of TNF-α in offspring from mothers with periodontitis may confirm previous evidence that low-grade systemic inflammation develops early in the process of T2DM [127].

Low-grade systemic inflammation caused by maternal periodontitis may contribute to numerous disease states, not limited to T2DM. A study by Pimentel et al. [121] shows that perinatal periodontal disease reduces social behavior in male offspring, thus is a risk factor for the development of central nervous system (CNS) disorders. However, it is worth mentioning that, in this study, no differences were observed in the frontal cortex level of the reelin-neuronal marker, contributing to the early neurodevelopmental pathology involved in schizophrenia and autism. Studies carried out on animal models that have detected that the learning, function, and memory of manic eagerness significantly affect male and female rats after trials of female rats to perform LPS (lipopolysaccharide) in controls with control offspring. This indicates that pre-pregnancy exposure to LPS Pg may intergenerally affect some behavioral functions in male and female offspring [128].

Epidemiological studies have indicated an association between maternal infection during pregnancy, and an increased risk of CNS disorders in offspring, including schizophrenia, autism, and cerebral palsy. Due to maternal inflammation, the concentration of pro-inflammatory mediators such as cytokines, chemokines, antibodies, and acute-phase proteins increases in the maternal bloodstream, thus increasing the permeability of the placental barrier and the fetal blood–brain barrier, allowing the inflammatory mediators to enter the fetal brain. In the fetal central nervous system (CNS), these pro-inflammatory mediators are able to activate microglial cells that can further release pro-inflammatory cytokines (TNF-α, IL-1β and IL-6) and antibodies that aggravate neuroinflammation. Neuroinflammation in fetus brain may affect processes that are pivotal for normal brain development and maturation. Moreover, both epidemiological and experimental studies show that the effect of maternal inflammation on neurodevelopment in offspring depends on the timing of maternal infection [129]. Studies on the mice model revealed that, in mid-gestation, the elevation of pro-inflammatory cytokines in fetal brain was not accompanied by an increase in the mRNA expression levels of the corresponding genes; thus, the observed cytokine level in the fetus brain had a maternal origin. Unlike, in late gestation-induced elevations of cytokine proteins, concomitant increases in the relative expression of the corresponding genes were accomplished, suggesting the endogenous production of cytokines by the fetal brain [130]. Studies on the animal model with experimentally induced oral infection (periapical lesions) in mothers before pregnancy showed that rats exposed in all pregnancy to maternal oral inflammation had higher than normal concentrations of IL-6, IL-1β, and TNF-α in their serum and brain [131]. Recent studies on rats show that maternal periodontitis in pregnancy programs the immune response of the offspring. In this study, 30-day-old rat pups were injected with LPS, resulting in acute lung injury. In offspring from mothers with periodontitis, substantial changes in neutrofiles and leucocytes activity were observed. The authors of this study assumed that the programming effect of maternal periodontitis may also be related to an increase in the reactive oxygen-derived species, as the elevated gene expression of COX-1 and -2 in lungs was also observed in the exposed offspring after the administration of LPS [132].

### 6.2. Maternal Periodontitis as a Dysbiosis of Intrauterine Microenviroment

*P. gingivalis* is considered a “keystone pathogen” in the pathogenesis of periodontitis. These bacteria are the most frequent microorganisms present during bacteremia in patients with periodontitis [132], and they are also claimed to be the most common microorganisms in the amniotic fluid and placental tissue [133,134]. Whether these bacteria can be part of the placental microbiome is debatable, as there is no consensus regarding the existence of the placental microbiome in healthy full-term pregnancies [135]. Even though the concept of placental microbiome in healthy pregnancies turns out to be false, the results show that the composition of placenta pathogens is very similar to the human oral microbiome, firmly confirming the importance of maternal oral health in the healthy development of the fetus. However, as was mentioned above in the case of maternal periodontal disease, the translocation of *P. gingivalis* to the amniotic fluid and placenta was observed and connected with adverse pregnancy outcomes, such as preterm birth or low birth weight. It should be highlighted that environmentally induced changes in placental development are likely to affect intrauterine development in several ways. Any changes in the amount of nutrients available for the fetus, changes in the placental endocrine function, or any changes in the placental phenotype play a crucial role in intrauterine programming.

Although the molecular mechanisms by which *P. gingivalis* can induce adverse outcomes during pregnancy and affect newborns are not clear, Chopra et al. (2020) [136] proposed seven potential pathogenic mechanisms to be involved:

(1) Direct invasion, translocation, and injury to the fetal–placental unit/interface and maternal tissues,
(2) Persistence and survival within the fetal and maternal tissues and immune response evasion,
(3) Increased production of proinflammatory cytokines and shift in maternal–fetal immune response from Th2 to Th1 with the onset of Th17/T regulatory cell imbalance,
(4) Activation of the acute-phase response,
(5) Onset of polymicrobial dysbiosis and development of pathobiont species,
(6) Increased oxidative stress in the fetal and maternal tissue, and
(7) Increased fetal adrenal cortisone production and the onset of fetal stress [135].

A very recent study on the animal model by [137] showed another possible pathogenic mechanism by which *P. gingivalis* may affect low birth weight. The oral and intravenous administration of *P. gingivalis* to pregnant mice causes gestational weight gain in dams and lower body weight in fetuses. Microarrays revealed altered gene expression profiles in the liver and BAT of dams, suggesting the presence of inflammatory responses, immune responses, and altered metabolism. The alteration in the BAT phenotype, as well as changes in the expression of genes related to lipid metabolism in the liver (*Lpin1*, *Lpin2*, and *Lxra*), after *P. gingivalis* administration may result in metabolic disorders in the mother, and impaired fetal development.

### 6.3. Does the Maternal Periodontitis Change the Epigenome of the Offspring?

Emerging evidence shows that epigenetic processes may contribute to the pathophysiology of inflammatory processes [138]. Moreover, recent studies suggest that epigenetic alterations are possibly triggered by the host microbiota and environmental cues [139]. These epigenetic alternations (DNA methylation and histone modifications) affecting gene expression without changes in the nucleotide sequence, can have a long-term effect on the host’s immune homeostasis [140], and may negatively impact the development of chronic inflammatory diseases. In contrast, microRNAs are a class of small, noncoding RNAs that regulate gene expression at the posttranscriptional level. MiRNAs affect target messenger RNAs (mRNAs) by binding to the 3′ untranslated regions (3′-UTRs), and in consequence, downregulate the production of proteins.

Previous studies showed the upregulation of the DNA methylation marker (DNMT3b) in mice with the experimentally induced periodontitis induced by the systemic oral gavage with *P. gingivalis*, or with a combination of local (ligature) and systemic induction (*P. gingivalis* gavage + ligature) [141]. In periodontitis patients, methylation studies on peripheral leukocytes, directly related to the systemic immune status, revealed changes in the ZNF718, HOXA4, and ZFP57 genes, which are related to immune response regulation, and antigen processing and presentation [142]. Moreover, in biopsies of solid gingival tissue, differences in the methylation of genes involved in the wound healing (*ROBO2*, *PTP4A3*), cell adhesion (*LPXN*), and innate immune response (*CCL26*, *DNAJC1*, *BPI*) were observed.

Recent data suggest that the involvement of miRNA in the periodontitis is complex. Changes in miRNAs expression were previously observed in the gingival tissue of animals with periodontitis [143]. Different miRNAs are expressed as a response against bacterial-induced inflammation; moreover, miRNAs may contribute to the hyperinflammation or resolution of periodontal disease.

Thus, the question arises as to whether the periodontal disease of a pregnant mother may lead to changes in the epigenome of the offspring? To the best of our knowledge, based on in-depth database searches, there are no papers directly addressing maternal periodontal disease to epigenetic changes in offspring. However, there is a great number of data showing the effect of maternal inflammation or maternal bacterial infection on the formation of epigenetic marks in neonates as part of the adaptive stress response. Epigenetic features within the placenta have been implicated in the pathogenesis of preeclampsia (PE), which may be one of the consequences of chronic maternal periodontitis. Maternal periodontitis may result ina 3- to 5-fold increased risk for developing PE [117]. There is evidence showing that preeclampsia resulted in hypomethylation observed in the promoter region of 11b-hydroxysteroid dehydrogenase type 2 (HSD11B2) in cord blood samples from neonates exposed to PE [144]. In addition, decreased methylation was reported for insulin-like growth factor 2 (IGF2) in differentially methylated regions, important for the gene regulation of imprinted genes [144]. A genome-wide promoter-based differential methylation (DM) analysis reveals that genes in the farnesoid X receptor and liver X receptor (FXR/LXR) pathway are enriched, indicating the dysfunction of lipid metabolism in cord blood cells. Additional biological functional alterations involve inflammation, cell growth, and hematological system development [145]. These findings indicate changes to immune response and predisposition to cardiovascular disorders in adult life in newborns from PE mothers.

Taking into account the pathophysiology of periodontitis, the role of miRNAs in maternal–fetal communication should be discussed. One of the newly proposed mechanisms of this miRNAs crosstalk might be exosomes. The exosome is a 50–120 nm-sized extracellular vesicle (EVs) released by a variety of cells, carrying donor protein, cytokines, and nucleic acid, such as RNA and miRNAs [146,147]. Exosomes ensure persistent communication between the mother and fetus. They participate in all stages of pregnancy, including embryogenesis, implantation, and parturition. Exosomes are released by both maternal and fetal cells, and carry various cargo loads that are reflections of the physiologic and metabolic state of the cells, as well as pathological conditions at the time of their release. Until recently, it was thought that communication between mother and fetus via exosomes took place only through information sent by the fetal cells to the mother’s cells, and thus serum exosomal miRNAs may be used as molecular biomarkers for the prenatal diagnosis of fetal disorders [148]. However, the latest evidence shows that maternal exposure to various risk factors during pregnancy, specifically those causing oxidative stress and inflammation, can generate exosomes with distinct cargo proteins that can cause a fetal inflammatory response through the delivery of pro-inflammatory mediators [149].

Whether maternal exosomal miRNAs can also be transferred to the fetus is not confirmed. However, it was previously reported that maternal diabetes affect the miRNAs profile in fetal circulation, in human umbilical vein endothelial cells (HUVEC) and placenta [150].

Previous studies have found that, in the pathogenesis of periodontitis, a subtype of periodontal MSCs called periodontal ligament stem cells (PDLSCs) can regulate the immune response via communication through exosomes. It was shown that, in the normal or inflammatory periodontal microenvironment, miR-155-5p enriched in exosomes of PDLSCs regulated the expression of SIRT1 protein in CD4+ T cells, thereby affecting the balance of T helper cells’ 17/regulatory T cell, which induces immune-mediated tissue destruction and chronic periodontitis [151]. There is also evidence demonstrating that miR-155-5p plays an essential role in the regulation of allergen-induced inflammation, in the pathogenesis of chronic skin allergy, and airway inflammation. MicroRNA-155-5p is also upregulated in an asthma model [152]. Furthermore, previous animal studies showed that maternal periodontitis increased the development of lung inflammatory allergic response in the offspring. Interestingly, the placental expression of miR-155-5p was also found in intrauterine growth restriction. When samples of saliva and blood plasma from periodontitis patients were examined for the profile of miRNAs, it resulted in the selection of eight miRNAs down-regulated in both types of samples (miR-103a-3p, miR-126-3p, miR150-5p, miR-199a-5p, miR-4485-5p, miR-6088, miR-6821-5p) [153]. Changes in the expression of mirR-103a-3p and miR-126-3p were also observed in insulin resistance and diabetes [154], and in the peripheral white blood cells of women whose pregnancies were complicated by the preterm prelabor rupture of membranes, or spontaneous preterm birth [155]. miR-103a-3p was down-regulated in placentas from pregnancies with preeclampsia [156]. Moreover, its exosomal expression from placental trophoblast increases due to exposure to glucose concentration [157].

## 7. Clinicaltrials.Gov Analysis

To provide an insight into clinical trials linking the topic of periodontal disease and pregnancy, the registry Clinicaltrials.gov (access 21 December 2021) was analyzed. The terms ‘pregnancy and periodontal disease’ were used during searching. Then, 28 studies were founded. Fifteen had their status defined as ‘completed’, 7 ‘unknown’, 2 ‘terminated’, 1 ‘withdrawn’, 1 ‘recruiting’, 1 ‘active, not recruiting’, and 1 ‘not yet recruiting’. After, the completed studies were carefully analyzed and included, if they were associated with the topic.

In the group of completed studies, one of them focused on the relationship between non-surgical periodontal therapy during pregnancy, and the levels of cytokines in gingival crevicular fluid and salivary stress-related hormones [NCT03336957]. Another investigated the level of interleukins during 2/3 trimester, and after delivery [NCT03449186]. The NCT01812083 study analyzed the polymorphism of the IL-1, receptor antagonist gene and adverse pregnancy outcomes in the Turkish population. It is known that active PD may stimulate the production of proinflammatory cytokines (mediated by T and B cells) and the activation of osteolytic pathways, which influence many systemic diseases [158,159,160,161]. Knowing that the higher interleukin levels stimulated by PD may enhance the risk of preterm delivery in pregnant patients [162], the effective treatment and prevention of PD during pregnancy should be treated as an essential issue.

Moreover, another clinical trial tried to assess the impact of periodontal condition on low birth delivery incidence [NCT02613468, NCT00641901] or preeclampsia [NCT00855504, NCT03088228]. The NCT00893802, NCT01549587, and NCT00097656 studies prospectively investigated the impact of PD treatment on pregnant adverse events, whereas in the NCT00490165 study, a retrospective analysis was conducted. Currently, the correlation of PD with the incidence of adverse pregnancy events seems to be certain. However, the published meta-analyses faced the problem of important confounders and biased results [163,164]. New, appropriately planned, and reported studies are needed. Although several studies were conducted, the effectiveness of PD treatment during pregnancy is still debatable. Some clinical trials report a lack of association between non-surgical treatment and a decrease in adverse pregnancy outcomes during pregnancy [56,114,165], whereas others confirm it [166,167].

The NCT04315532 trial evaluated the impact of periodontal treatment on estrogen and dehydroepiandrosterone (DHEA) levels, whereas the NCT01422122 study assessed the role of vitamin D supplementation. It was shown that DHEA levels correlated with PD severity [168]. Non-surgical treatment did not influence DHEA levels [169]. The importance of estrogen deficiency and the impact of treatment on its level in human PD are poorly described in the literature. Vitamin D deficiency is reported as the risk factor of PD, and its supplementation may be beneficial in PD outcome [170,171]. In addition, the administration of vitamin D seems to be promising in reducing the risk of adverse pregnancy outcomes [172,173,174]. However, its efficiency should be confirmed.

Although we presented many pieces of evidence supporting the role of epigenetics in PD and pregnancy outcomes, there is a lack of clinical trials analyzing both conditions and epigenetic changes in the one model. Studies like this should be conducted in the future. As Kajiya et al. declared, future research should lead to the development of new preventive and therapeutic strategies, in order to make the outcome of patients with PD better [158].

**Figure 1:**
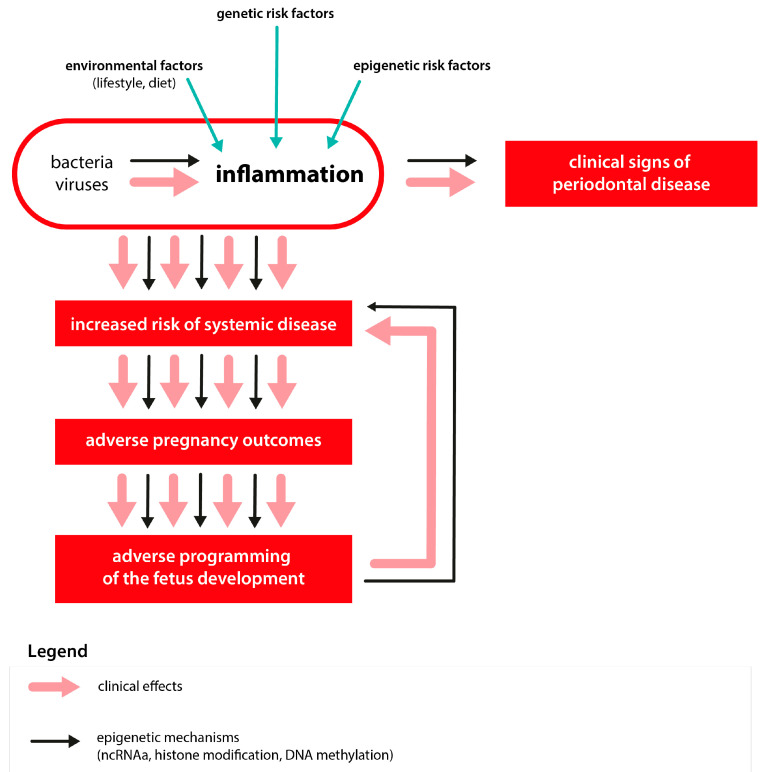
Relationship between periodontal disease in pregnant women and epigenetically programmed general diseases in the offspring.

The literature review presented above indicated the existence of significant relationships between the occurrence of PD in mothers, and the appearance of epigenetically programmed general diseases in their offspring. These processes are schematically shown in Figure 1.

## 8. Conclusions

Periodontitis is a complex disease. Previous epidemiologic and experimental studies have shown that it may also impact several systemic diseases, whereas periodontal treatment leads to an improvement of glycaemic control in patients with type 2 diabetes [175], and metabolic syndrome [176], as well as improved renal function associated with diabetes [177]. Maternal periodontal disease also leads to an increase of preeclampsia and preterm births, as well as cardiovascular [144,145] disease, allergies, and asthma in the offspring [151,152]. Several mechanisms and pathological pathways have been linking periodontitis with these disorders. Moreover, a significant association between periodontal disease in pregnancy and short-term and long-term adverse outcomes for offspring was found in multiple studies, and suggested in many reviews. Although the pathological mechanisms which impact fetus development in the course of maternal periodontal changes are complex, it seems plausible to consider the inclusion of periodontal treatment in a group of recommended procedures for preparing for pregnancy. Obstetricians should inform women who are planning to conceive that it might be beneficial to the outcome of the future pregnancy and long-lasting child health, to receive periodontal examination and treatment before becoming pregnant. Moreover, in pregnancy periodontal treatment provided to mothers with mild to moderate periodontitis, when done before the 21st week of gestation, it was shown to prevent premature births [177]. Pregnant women should be aware of the periodontal changes associated with pregnancy, and that periodontal therapy during pregnancy is effective and safe, both for them and for the fetus.

## Data Availability

The data presented in this study are available on request from the corresponding author.
